# Differentially expressed proteins and microbial communities of the skin regulate disease resistance to Chinese tongue sole (*Cynoglossus semilaevis*)

**DOI:** 10.3389/fimmu.2024.1352469

**Published:** 2024-04-22

**Authors:** Lei Wang, Min Tian, Songlin Chen

**Affiliations:** ^1^ State Key Laboratory of Mariculture Biobreeding and Sustainable Goods, Yellow Sea Fisheries Research Institute, Chinese Academy of Fishery Sciences, Qingdao, Shandong, China; ^2^ Laboratory for Marine Fisheries Science and Food Production Processes, Laoshan Laboratory, Qingdao, Shandong, China; ^3^ Shandong Key Laboratory of Marine Fisheries Biotechnology and Genetic Breeding, Yellow Sea Fisheries Research Institute, Chinese Academy of Fishery Sciences, Qingdao, Shandong, China; ^4^ Shandong Provincial Key Laboratory of Animal Resistance Biology, College of Life Sciences, Shandong Normal University, Jinan, Shandong, China

**Keywords:** *Cynoglossus semilaevis*, skin, proteome, microbial community, protein microbial interaction

## Abstract

Vibriosis, caused by *Vibrio*, seriously affects the health of fish, shellfish, and shrimps, causing large economic losses. Teleosts are represent the first bony vertebrates with both innate and adaptive immune responses against pathogens. Aquatic animals encounter hydraulic pressure and more pathogens, compared to terrestrial animals. The skin is the first line of defense in fish, constituting the skin-associated lymphoid tissue (SALT), which belongs to the main mucosa-associated lymphoid tissues (MALT). However, little is known about the function of immunity related proteins in fish. Therefore, this study used iTRAQ (isobaric tags for relative and absolute quantitation) to compare the skin proteome between the resistant and susceptible families of *Cynoglossus semilaevis*. The protein integrin beta-2, the alpha-enolase isoform X1, subunit B of V-type proton ATPase, eukaryotic translation initiation factor 6, and ubiquitin-like protein ISG15, were highly expressed in the resistant family. The 16S sequencing of the skin tissues of the resistant and susceptible families showed significant differences in the microbial communities of the two families. The protein-microbial interaction identified ten proteins associated with skin microbes, including immunoglobulin heavy chain gene (IGH), B-cell lymphoma/leukemia 10 (BCL10) and pre-B-cell leukemia transcription factor 1 isoform X2 (PBX2). This study highlights the interaction between skin proteins and the microbial compositions of *C. semilaevis* and provides new insights into understanding aquaculture breeding research.

## Graphical Abstract

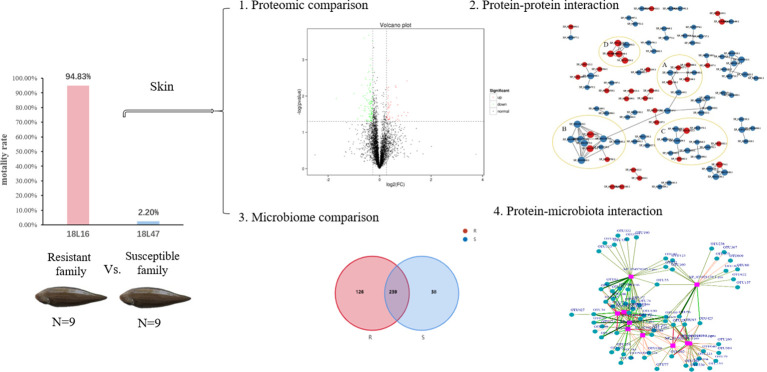

## Highlight:

- Proteome profiling reveals skin composition of Chinese tongue sole.
- The resistant family exhibits 464 highly expressed proteins and 400 down regulated proteins.
- Protein-protein interaction analysis reveals 14 high expressed proteins in the resistant family.
- Ten proteins show associations with skin bacteria.

## 1 Introduction

The skin is a complex and dynamic ecosystem inhabited by bacteria, archaea, fungi, and viruses (1). Skin cells, immune cells and microbes maintain the physical and immune barrier under homeostatic and healthy conditions and various stress conditions, especially infection or wounding (2). Skin immunity and physiology homeostasis are closely related to skin microbiota. Fish skin is a vital organ that serves many functions, including mechanical protection, homeostasis, osmoregulation, and protection against diseases. The expression of skin proteins changes under different pathological conditions. Fish skin contains molecules with immunologically important properties, and interact directly with commensal microbial populations on the mucosal surfaces (3). Skin, gill and gut are part of the fish mucosal immunity, and the mucosal immunology of teleost fish has received much attention because of its key role as the first barrier against infection. During infection, the skin is the main target of bacteria and the first line of defense against invading pathogens, making it the initiator of the adaptive and innate immune responses that protect against physical damage and pathogen infection.

The prevalence of vibriosis is one of the most serious factors affecting marine fish. Fish rely on the immune response, including innate and adaptive immunity, to protect against vibriosis caused by *Vibrio* colonization and subsequent skin breaching or through the gut. After breaching the outer barrier, the infectious agent evades the host’s defense mechanisms through several mechanisms and establishes a severe systemic infection (4). Transcriptomic and proteomics analyses have been performed to identify multiple genes involved in immunity during *Vibrio* infection in Chinese tongue sole (*Cynoglossus semilaevis*), turbot (*Scophthalmus maximus*), and orange-spotted groupers (*Epinephelus coioides*) (5–8).

The Chinese tongue sole (*Cynoglossus semilaevis*) is a high-value marine flatfish cultured in China, especially in Hebei, Shandong, and Fujian provinces, with annual output value of more than two billion yuan. Various bacteria and viruses cause severe economic losses of Chinese tongue sole. *Vibrio harveyi* is a Gram-negative bacteria that affects economic fish, shellfish, shrimps, and sea cucumbers worldwide. *V. harveyi*-infected Chinese tongue sole shows epidemic vibriosis with symptoms of serious skin ulceration and hemorrhage septicemia (9). Most previous reports on anti-bacterial mechanisms focused on the visceral tissues, such as spleen, head kidney, intestine, and liver.

This study conducted skin proteomics to characterize the proteins and microbiota present in the skin of the disease-resistant and susceptible fish families using iTRAQ (isobaric tags for relative and absolute quantitation) assay. The main functional proteins were also identified to determine the mechanisms underlying fish disease resistance. Overall, this study demonstrates the association between anti-disease-related skin proteins in Chinese tongue sole, and could be explored further to understand of the molecular mechanisms of host-pathogen interactions in *C. semilaevis*.

## 2 Materials and methods

### 2.1 Fish rearing and sample collection

The families of *C. semilaevis* were produced in the aquaculture base of Yellow Sea Fisheries Research Institute. The challenge test was conducted in 57 families with *Vibrio harveyi* using a median lethal dose (LD_50_) by intraperitoneal injection (2.5 × 10^6^ cfu/per 5 g body weight). The dead fish were counted and removed daily at an of eight hours, and mortality was recorded for 14 days. The survival data was analyzed as described previously (10). After challenge test, eighteen fish belonging to two families were obtained from the uninfected fish, as the resistant family (F18L16, 9 fish, survival rate: 94.83%) and the susceptible family (F18L47, 9 fish, survival rate: 2.20%). The fish (weighing 122.5 ± 3.7 g) were reared in square tanks at 20 ± 2°C with a continuous seawater supply. After acclimation for 7 days, the fish were anesthetized with a lethal dose of MS-222 (300ppm). The skin tissues (including epidermis and dermis) were then collected from the two families and immediately frozen in liquid nitrogen for proteome and microbiome studies, and RNAliter (Solarbio, Beijing, China) for RNA extraction.

### 2.2 Protein digestion, iTRAQ labeling and fractionation

Every three samples were mixed at equal weight to reduce individual differences, and each families contained three biological replicates. Skin tissue (50 mg) from the resistant and susceptible families was transferred into a 2 mL centrifuge tubes on ice, and added 500 μL of the 1 ×cocktail with of SDSL3 and ethylenediaminetetraacetic acid (EDTA) for 5 min, after which dithiothreitol (DTT) was added to a final concentration of 10 mM. Two 5 mm magnetic beads were added into the centrifuge tubes for high-speed tissue lysis via centrifugation at 25000 g for 15 min at 4°C. The supernatant was collected and precooled acetone (5 times the volume) was added, following by incubation at -20°C for 2 h. The mixture was centrifuged at 25 000 gfor 15 min at 4°C, and the supernatant was discarded, after which the precipitate was air-dried. An appropriate amount of lysis buffer without SDS was added to dissolve the protein precipitate, followed by centrifugation at 25000 g for 15 min at 4°C to collect the supernatant. After that, 10 mmM of DTT was added, and the mixture was incubated at 56°C in the water bath for 1 h, followed by the addition of 55 mM IAM for incubation in the dark for 45 min. Precooled acetone (five times the volume) was then added, and the mixture was incubated at -20°C for 2 h, followed by centrifugation at 25000 g for 15 min at 4°C. The supernatant was discarded and the precipitate was air-dried, dissolved in lysis buffer without SDS, and centrifuged (25000 g for 15 min at 4°C) to collect the supernatant. The supernatant was used for total protein quantification via Bradford assay, as previously described (11).

### 2.3 iTRAQ labeling and analysis

The iTRAQ labeling and assay were performed as previously described (11) but with some modifications. Briefly, protein samples were incubated with 10 μL of 1 mg/mL Trypsin Gold (Promega, Madison, WI, USA) at 37°C for 16 h. The proteins from the resistant and susceptible families were labeled with 111 to 119 tags of the iTRAQ reagents, respectively. Then, the peptides were labeled with the isobaric tags, incubated at room temperature for 2 h, and dried by vacuum centrifugation. The labeled samples were pooled and purified using a strong cation exchange chromatography (SCX) column (Phenomenex, Torrance, CA, USA). The purified samples were separated by liquid chromatography (LC) on an LC-20AD nanoHPLC system (Shimadzu, Japan) using the auto-sampler onto a 2 cm C18 trap column (Code. 186002574, Waters, America). Data acquisition was performed on a Triple TOF 5600 System fitted with a Nanospray III source (AB SCIEX) and a pulled quartz tip as the emitter (New Objectives, MA). The iTRAQ data were analyzed using MASCOT 2.3.02 software and protein identification was performed using the Chinese tongue sole genome database (Bioproject PRJNA73987).

### 2.4 GO, KEGG and PPI analysis

Proteins with a statistically significant ratio of the fold change value of ≥1.2 and a p-value of <0.05 were considered differentially expressed protein (DEPs). Functional annotations of the DEPs were conducted using the Blast2GO program against the non-redundant protein database (NR, NCBI). Hypergeometric tests with a ratio of p < 0.05 were used to identify DEPs in the gene ontology (GO) enrichment terms. The proteins were subjected to functional annotation analysis according to the COG (http://www.ncbi.nlm.nih.gov/COG/) and KEGG databases (http://www.genome.jp/kegg/). Pathways significantly enriched with DEPs with p < 0.05 and a false discovery rate (FDR) of <0.05 were used as a threshold to select significant KEGG pathways. The online tool STRING (http://www.string-db.org) was used to construct the protein-protein interaction (PPI) maps to further investigate the relationship between immune-related genes and discover the major resistance proteins.

### 2.5 Skin macrobiotic sequencing

The DNA samples of resistant and susceptible families (N=9) were extracted according to the protocol of the marine animal DNA extraction kit (Tiangen, Beijing, China). DNA integrity and purity were determined using 1% agarose gel electrophoresis. Specific primers with barcodes were synthesized, and PCR was conducted. The amplified products were purified, quantified and homogenized to form a sequencing library (SMRT Bell), and the library quality inspection was conducted. The qualified library was sequenced using the PacBio Sequel system. The off-board data from the PacBio sequel were in bam format, and the circular consensus sequencing (CCS) files were exported by Smrtlink analysis software. The data from different samples were classified according to the barcode sequence and were converted into fastq format.

### 2.6 Sequencing data processing

All statistical analyses were conducted using R software on the BMKCloud platform (www.biocloud.net). The sequencing reads were classified into operational taxonomic units (OTUs), which contained sequences with ≥97% similarity. The OTU annotations were based on the Silva (Silva_119_release_aligned) database (12). Microbial α‐diversity estimates (abundance-based coverage estimate [ACE], and Chao1 and Shannon indexes) were calculated based on the OTU assignment (13). The relative abundance (i.e., the proportion of sequences from a phylum/class relative to the total number of sequences in the sample) was calculated. A heatmap analysis was performed using drawing tools. The generation of rarefaction curves and the hierarchical clustering analysis, and principal coordinate analysis (PCoA) were performed using Past3 software (Version 3.22).

### 2.7 Relationship analysis between iTRAQ data and microbiota

The relationship between iTRAQ data and microbial communities was analyzed by correlation test using redundancy analysis (RDA; Canoco 5) and the STRING database. The analysis was performed on the BMKCloud platform (www.biocloud.net). Microbiome (OTU level) using t-test method and the threshold was p-value <0.01. The differentially expressed protein was screened using t-test; the threshold was FC>1, p-value<0.05. The interaction pairs of the target proteins and OTUs were directly extracted from the database to construct the interaction network using igraph in R package, and imported into Cytoscape software for visualization.

### 2.8 Real-time quantitative PCR of the ten disease resistance-related proteins

The primers for DEPs used for the RT-qPCR were designed using NCBI primer BLAST, and *β-actin* gene was selected as the reference gene. Total RNA from skin were extracted using the TRIzol reagent (Invitrogen) according to manufacturer’s instruction and reverse-transcribed for first-strand cDNA synthesis in a total reaction volume of 20 μL. RT-qPCR was performed using 10 μL of SYBR qPCR Master Mix TSE501 (Qingke Biotech Co., Ltd.), 2 μL of cDNA, 0.5 μL of each primer pair, and 7 μL ddH_2_O. The amplification conditions were denaturation at 95°C for 1 min, followed by 40 cycles of denaturation at 95°C for 10 s and annealing at 60°C for 30 s. The experiment was conducted in triplicate, and the relative gene expression were analyzed using the delta-delta Ct (2^-ΔΔCt^) method.

## 3 Results

### 3.1 iTRAQ analysis of skin protein profiles

**Figure 1:**
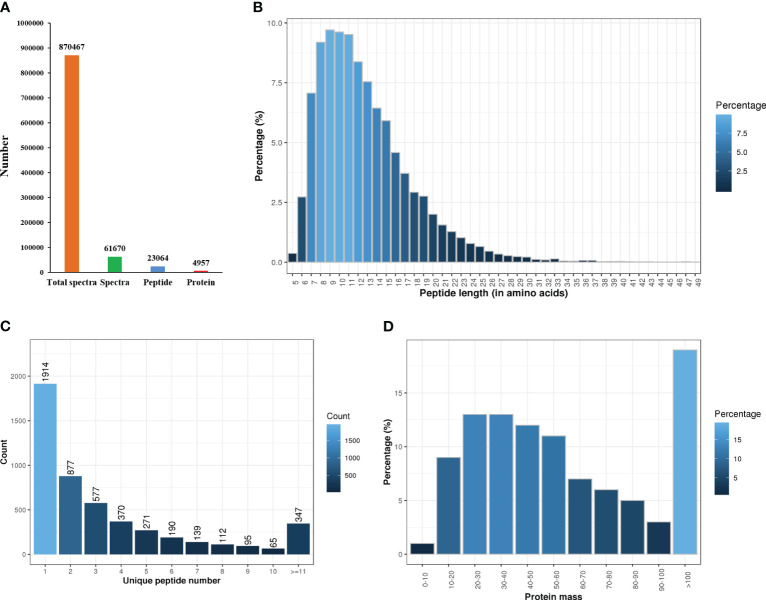
Statistics of proteome sequencing and annotation. **(A)** Total number of spectra, peptides and proteins. **(B)** Peptides length. **(C)** The number of unique peptides. **(D)** Protein mass.

The protein concentration of the six samples ranged from 5.16-8.14 μg/μL, with a protein volume of 250 μL and a total protein concentration of 1288.95-2035.88 μg. The iTRAQ analysis of *C. semilaevis* skin proteome generated 870467 raw spectra (including 61670 spectra and 23064 peptides), and identified 4957 proteins in Mascot (**Figure 1A**). The lengths of most peptides ranged from 7-15 amino acids, with the maximum percentage of peptides being 9 amino acids long (**Figure 1B**). Among the 4957 identified proteins, 1914 had one peptide, and 374 had more than 11 peptides (**Figure 1C**). The molecular weights of 50% of the proteins ranged from 20 to 60 kDa, while more than 18% had molecular weights of more than 100 kDa (**Figure 1D**).

### 3.2 Identification of differentially expressed proteins (DEPs)

**Figure 2:**
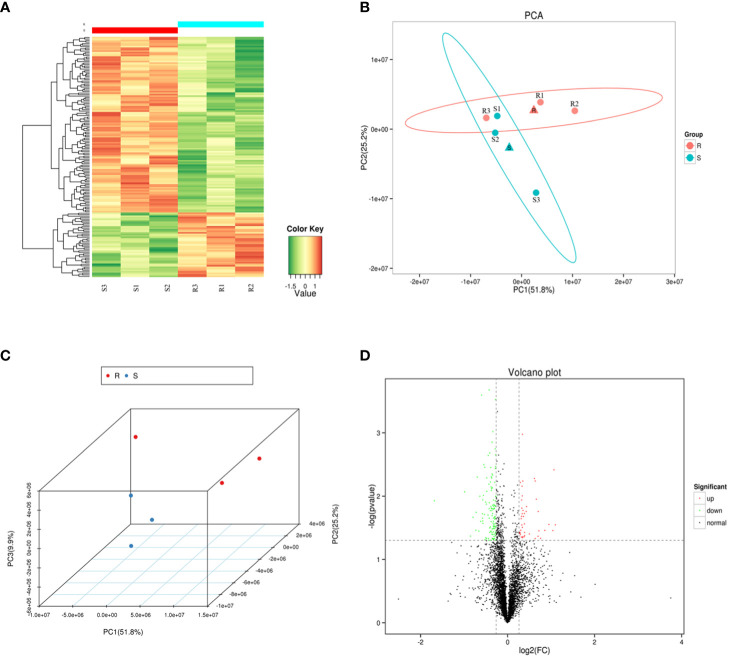
The heat map, principal component analysis (PCA) analysis and volcano plot of the six samples. **(A)** A heat map of the top 200 differentially expressed proteins (DEPs) identified via isobaric tags for relative and absolute quantitation (iTraq) analysis. **(B–C)** Principal component analysis (PCA) in 2D and 3D models of DEPs. **(D)** Volcano plot showing up regulated (red color dots) and down regulated (green color dots) proteins between the two groups.

A heat map, PCA and volcano plot revealed distinct protein expression patterns of the DEPs between the resistant and susceptible families (**Figure 2**). The heat map identified the top 200 DEPs via iTraq analysis (**Figure 2A**), and the two groups were separated into two distinct clusters in PCA 2D and 3D models (**Figure 2B** and **2C**). 398 upregulated and 475 downregulated proteins were detected in volcano plot (**Figure 2D**).

### 3.3 GO and KEGG enrichment analyses of DEPs

**Figure 3:**
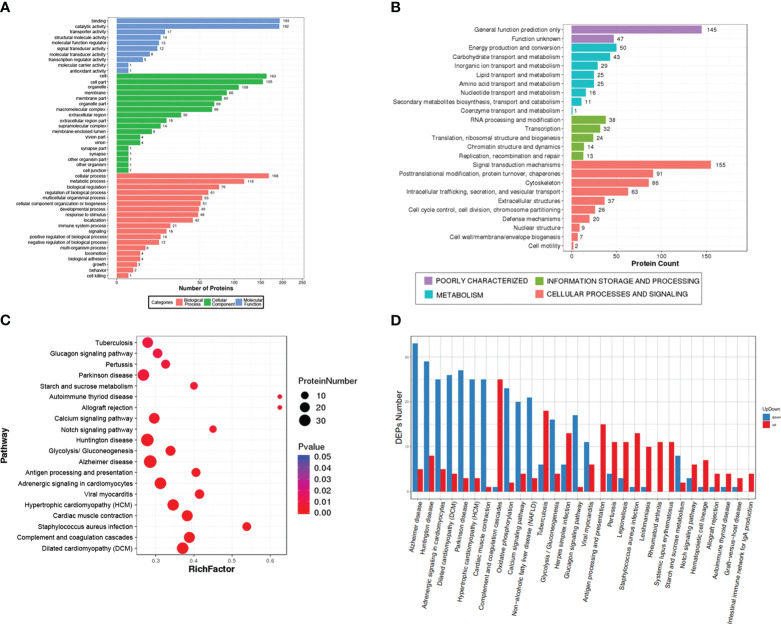
Gene Ontology (GO), Eukaryotic Orthologous Groups (KOG) and Kyoto Encyclopedia of Genes and Genome (KEGG) analysis of the differentially expressed proteins (DEPs). **(A)** The GO term categories are based on biological processes, cellular components, and molecular function. **(B)** KOG classification of DEPs. **(C)** The top 20 signal pathways in the KEGG enrichment. **(D)** the number of upregulated and downregulated proteins in the top 30 signal pathways.

A total of 873 DEPs, including 398 up-regulated and 475 down-regulated, were enriched in 47 GO terms and classified into three grade I functional components according to GO enrichment analysis. These GO terms included biological process (19 terms), cellular component (18 terms) and molecular function (10 terms) (**Figure 3A**). The DEPs were used to search against the KOG database and were enriched in 25 KOG terms, including signaling transduction mechanisms (protein number: 155), posttranslational modification, protein turnover, chaperones (protein number: 91), cytoskeleton (protein number: 86), intracellular trafficking, secretion, vesicular transport (protein number: 63), and defense mechanisms (protein number: 20) (**Figure 3B**). The top 20 pathways included some immune-related pathways (complement and coagulation cascades and antigen processing and presentation) (**Figure 3C**). The upregulated protein and downregulated proteins in these pathways are showed in **Figure 3D**, and they include complement and coagulation cascades (up: 25, down: 1), antigen processing and presentation (up: 15, down: 0), and intestinal immune network of IgA production (up: 4, down: 0).

### 3.4 PPI network analysis of the hub proteins

**Figure 4:**
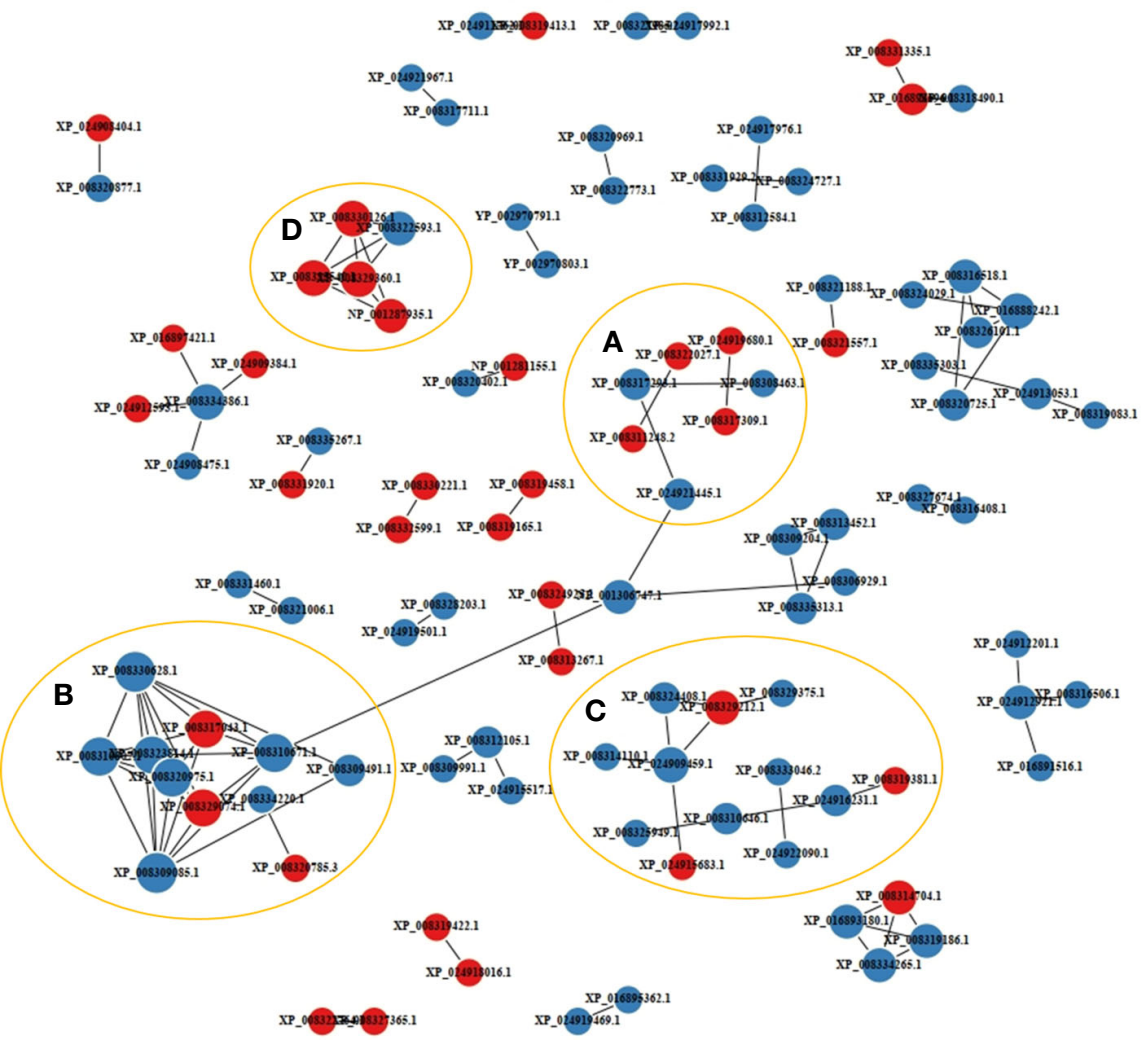
Protein-protein interaction network of the disease resistance-related differentially expressed proteins (DEPs). The labels with red and blue circles indicate upregulated and downregulated DEPs, respectively. A-D represented interacting protein clusters.

A PPI network analysis was conducted using 107 proteins to reveal the relationship between the resistance-related DEPs (**Figure 4**). Four closely related protein interaction clusters were found (**Table 1**), and in which 14 proteins had high expression in the resistant family. These proteins included integrin alpha-5-like isoform X3, integrin beta-2, protein S100-A1-like, and the heat shock protein HSP 90-beta isoform X1 in Cluster A; the alpha-enolase isoform X1, neuron navigator 1 isoform X5, and V-type proton ATPase subunit B, brain isoform-like in Cluster B; U6 snRNA-associated Sm-like protein LSm7, eukaryotic translation initiation factor 6, and ubiquitin carboxyl-terminal hydrolase 4 isoform X3 in cluster C; 26S proteasome non-ATPase regulatory subunit 4, neuroligin-3 isoform X2, ubiquitin-like protein ISG15, and proteasome subunit beta type-2 in cluster D. These proteins may play significant roles in regulating disease resistant in fish. However, some proteins, such as citrate synthase, aspartate aminotransferase, and phosphoglycerate kinase 1, may negatively regulate antibacterial activities.

**Table 1:** Proteins in the four clusters of the protein-protein interaction network.

Cluster A		
Color	Protein ID	Protein name
Red	XP_008317309.1	integrin alpha-5-like isoform X3
	XP_024919680.1	integrin beta-2
	XP_008322027.1	protein S100-A1-like
	XP_008311248.1	heat shock protein HSP 90-beta isoform X1
Blue	XP_008317293.1	citrate synthase, mitochondrial
	XP_008308463.1	aspartate aminotransferase, mitochondrial
	XP_024921445.1	dihydrolipoyllysine-residue acetyltransferase component of pyruvate dehydrogenase complex, mitochondrial isoform X2
Cluster B		
Color	Protein ID	Protein name
Red	XP_008317043.1	alpha-enolase isoform X1
	XP_008319074.1	neuron navigator 1 isoform X5
	XP_008320785.1	V-type proton ATPase subunit B, brain isoform-like
Blue	XP_ 008330628.1	phosphoglycerate kinase 1
	XP_008310807.1	bisphosphoglycerate mutase
	XP_008323814.1	beta-enolase
	XP_008310671.1	pyruvate kinase PKM-like
	XP_008320975.1	glyceraldehyde-3-phosphate dehydrogenase
	XP_008334220.1	ATP synthase subunit alpha, mitochondrial
	XP_008309491.1	probable fructose-2,6-bisphosphatase TIGAR A
	XP_008309085.1	glucose-6-phosphate isomerase
Cluster C		
Color	Protein ID	Protein name
Red	XP_008329212.1	U6 snRNA-associated Sm-like protein LSm7
	XP_008319381.1	eukaryotic translation initiation factor 6
	XP_024915683.1	ubiquitin carboxyl-terminal hydrolase 4 isoform X3
Blue	XP_008324408.1	U4/U6 small nuclear ribonucleoprotein Prp4
	XP_008329375.1	U7 snRNA-associated Sm-like protein LSm10
	XP_008314110.1	ubiquitin carboxyl-terminal hydrolase 15 isoform X4
	XP_024909459.1	squamous cell carcinoma antigen recognized by T-cells 3-like
	XP_008333046.2	NADH dehydrogenase [ubiquinone] 1 alpha subcomplex subunit 11
	XP_008310646.1	RNA cytidine acetyltransferase
	XP_024916231.1	probable ATP-dependent RNA helicase DDX27
	XP_008325949.1	DNA-directed RNA polymerase I subunit RPA1
	XP_024922090.1	NADH dehydrogenase [ubiquinone] 1 alpha subcomplex subunit 13
Cluster D		
Color	Protein ID	Protein name
Red	NP_001287935	Ubiquitin-like protein ISG15
	XP_008325540	neuroligin-3 isoform X2
	XP_008330126	26S proteasome non-ATPase regulatory subunit 4
	XP_008329360	proteasome subunit beta type-2
Blue	XP_008322593	lys-63-specific deubiquitinase BRCC36

### 3.5 Specific OTUs between resistant and susceptible families

**Figure 5:**
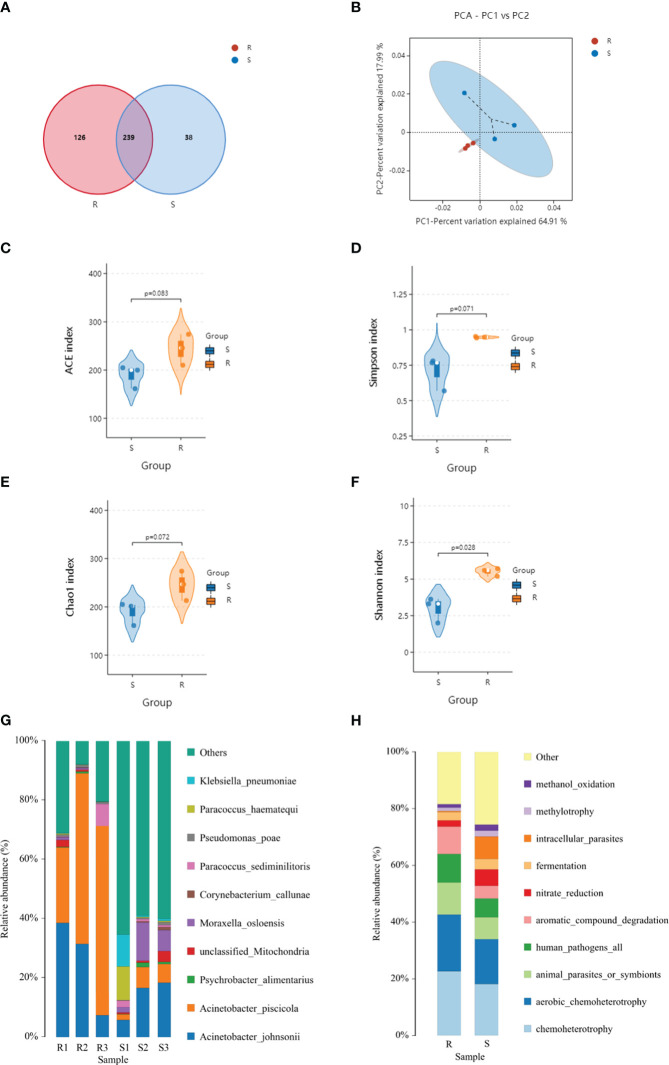
Venn diagram **(A)**, principal component analysis (PCA) **(B)**, species distribution **(C–F)**, and faprotax analysis **(G, H)** of the resistant and susceptible families.

A metagenomic analysis was conducted to determine the composition differences between the resistant and susceptible families and the results revealed a correlation between protein profiles and microbiota. Based on the high throughput-sequencing data of skin microbes, 420 OTUs were annotated, among which 126 were specific to the resistant family, and 38 were specific to the susceptible family. The common OTUs were 239, according to principal coordinates analysis (PCoA). In the resistant family, the top dominant phyla of the skin microbial community was Proteobacteria, followed by Firmicutes and Bacteroidota. However, the percentage of Actinobacteriota was upregulated in the susceptible family (**Figure 5**).

**Figure 6:**
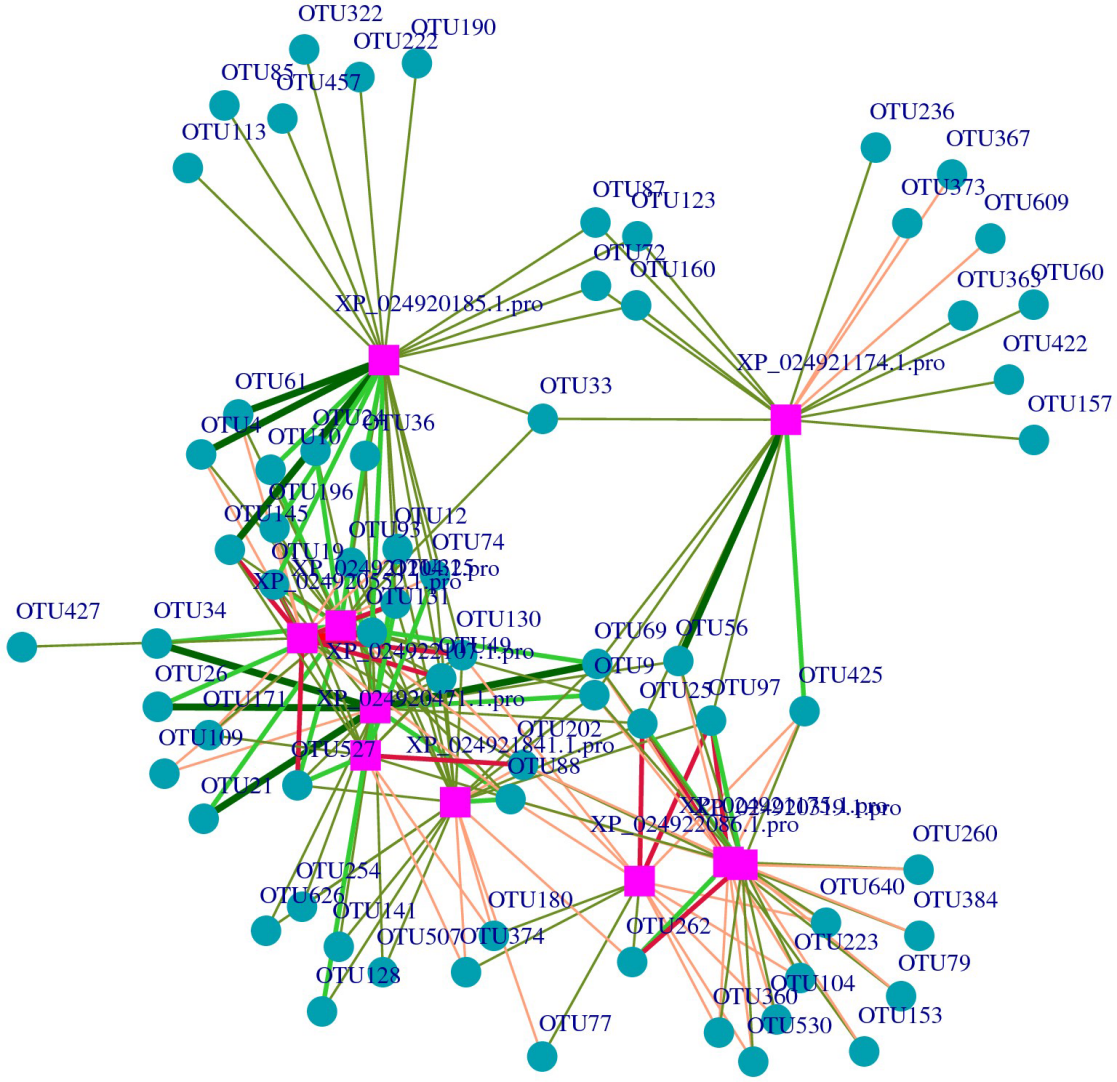
Network of the relationships between proteins with operational taxonomic units (OTUs). The purple squares represent the proteins, and the blue change to: circles represent the OTUs.

After screening, the differential metabolites were 377 and the DEPs were 339. The purple squares represent proteins, while the cyan cycles represent the 49 related operational taxonomic units (OTUs). The red lines indicate a positive correlation, while the green lines indicate a negative correlation. The width of the lines represents the level of correlation (**Figure 6**). In the network, 10 proteins related to skin microbes were detected. The proteins and related OTUs are listed in **Supplementary Tables S1**, **S2**.

### 3.6 RT-qCR analysis of the 10 proteins in the resistant and susceptible families

**Figure 7:**
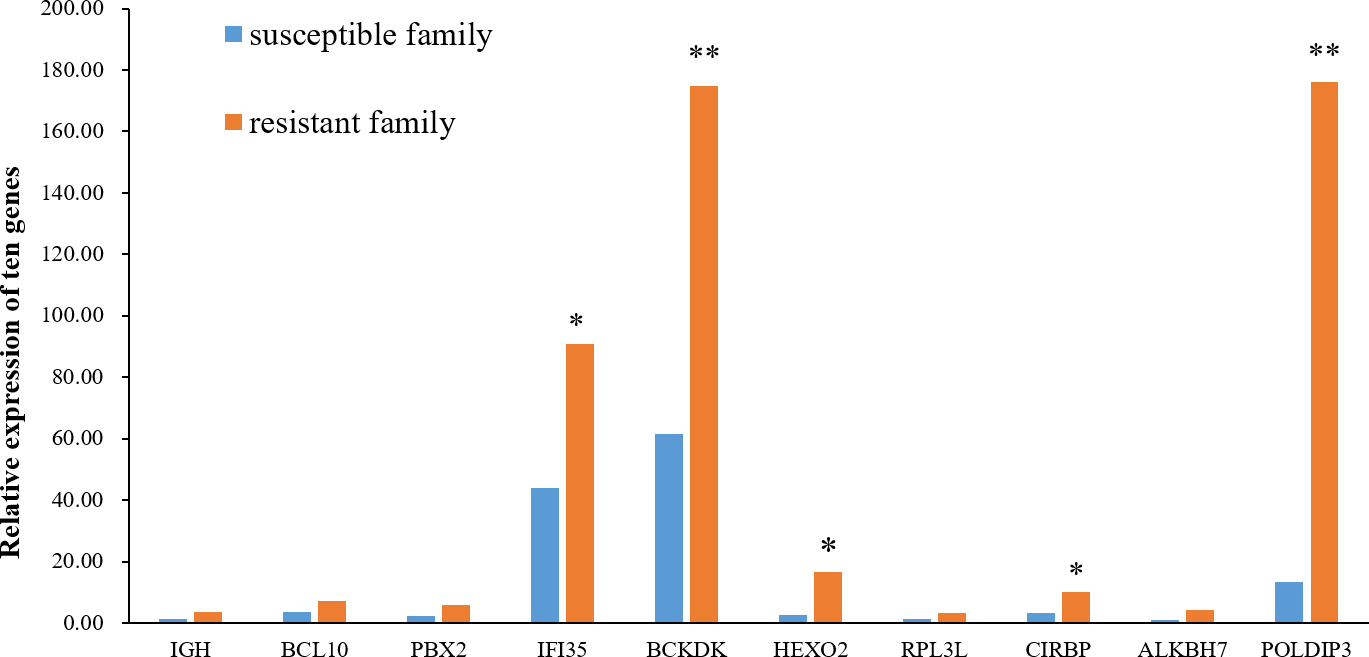
Relative expression of the ten hub genes in the skin of resistant and susceptible families. Three technical replicates were set for each biological replicate. The asterisks indicate statistical significance (*p < 0.05; **p < 0.01).

The relative mRNA expression levels of the ten hub proteins in resistant and susceptible families were analyzed via RT-qPCR. The results indicated that mRNA expression levels of most genes were showed a consistent tendency with proteins, and the transcript levels of IFI35, BCKDK, HEXO2, CIRBP, and POLDIP3 were relatively higher in the resistant family than in the susceptible family (**Figure 7**).

## 4 Discussion

Due to their aquatic environments, fish skin is an essential protective barrier and differs from the exposed skin of other vertebrates. The fish skin is a multifunctional organ involved in protection, communication, sensory perception, ion regulation, and excretion, making it an important first line of defense against the invasion of environmental pathogens (14). Additionally, as an important immune tissue, fish skin responds to attack by different pathogens by inducing the mucosal immune-related proteins. The transcriptional profiles and real-time quantitative PCR assays of the skin tissues of common carp (*Cyprinus carpio*), Atlantic salmon (*Salmo salar*) and Atlantic cod (*Gadus morhua*) identified many genes involved in antibacterial, antivirus and anti-parasite responses (15–19). A recent research found that a parasite co-infection with a virus or bacteria causes severe skin damage in wild and farmed Atlantic salmon. Cai et al. conducted a transcriptomic analysis of sea lice (*Lepeophtheirus salmonis*) and infectious salmon anemia virus (ISAv) co-infection of the Atlantic salmon (*Salmo salar*) skin. The study found that the up-regulated genes were associated with glycolysis, the interferon pathway, complement cascade activity, and heat shock protein family, while the down-regulated genes were related to antigen presentation and processing, T-cell activation, collagen formation, and extracellular matrix (15). Co-infection of sea lice (*Lepeophtheirus salmonis*) with formalin-killed *Aeromonas salmonicida* activated the pathways involved in the innate (e.g., neutrophil degranulation) and adaptive immunity (e.g., antibody formation), and endothelial cell migration (20).

Several high-throughput protein assays have been conducted in various fish to determine the molecular mechanism of fish skin as a pathogen barrier at the proteome-level. A previous study identified 17 proteins differentially expressed in zebrafish (*Danio rerio*) skin under *Aeromonas hydrophila* infection, and these included the DR α 1 domain of the MHC class II and the immunoglobulin heavy chain V-region (21). Another study on the differential proteomics of the zebrafish skin in response to SVCV infection examined the by isobaric tags for relative and absolute quantitation (iTRAQ). The study found that DEPs were significantly associated with complement, inflammation, and antiviral response (22). Furthermore, Tan et al. examined the differential proteomic profiling of the *V. vulnificus*-infected skin of Chinese tongue sole and found that 16 immune signal pathways were significantly enrich. These pathways included focal adhesion, adherens junction, phagosome, leukocyte transendothelial migration, complement and coagulation cascades, antigen processing and presentation, bacterial invasion of epithelial cells, calcium signaling pathway, ECM-receptor interaction, etc (23).

To identify the key genes involved in fish resistance, we performed transcriptomic analysis using a mixture of liver, kidney and spleen tissues of *C. semilaevis*, and compared the immune responses of resistant and susceptible families before and after *V. harveyi* infection. We identified 713 genes exhibiting significant differences at the transcript level, of which some such as lyg, tlr4, sdf2, cxcr4, tacr3, and others were more highly expressed in the resistant family than in the susceptible family (24). For skin tissues, 1421 differentially expressed genes were identified and were significantly enriched in ECM-receptor interaction, complement and coagulation cascades, cardiac muscle contraction, starch and sucrose metabolism and aminoacyl-tRNA biosynthesis pathway (25). These findings indicated that multiple genes might control fish resistance against vibriosis.

This study found that integrins, including integrin alpha-5-like isoform X3 and integrin beta-2 in Cluster A, were highly expressed in the resistant family. These integrins were enriched in ECM-receptor interaction (map04512), leukocyte transendothelial migration (map04670), bacterial invasion of epithelial cells (map05100), phagosome (map04145), cell adhesion molecules (map04514), focal adhesion (map04510), and tight junction (map04530) (https://www.kegg.jp/). Integrins αMβ2 and αXβ2 mediate macrophage recognition and phagocytosis of pathogenic bacteria and play a key role in the resistance against invasive pathogens (26). Integrin 4 reportedly prevented *V. alginolyticus* infection and regulated phagocytosis (as a cell adhesion receptor) of oyster *Crassostrea hongkongensis* (27). The alpha-enolase in cluster B is a part of a dimeric enzyme of the glycolytic pathway responsible for catalyzing the conversion of 2-phosphoglycerate to phosphoenolpyruvate (28). The subunit B of the V-type proton ATPase is a part of v-type ATPase and plays a major role in ion regulative processes in the apical membranes of fish skin and gills (29, 30). The eukaryotic translation initiation factor 6 (eIF6) in cluster C is one of the important initiation factors that play an important role in both ribosome biogenesis and protein translation. The eIF6 interacted with Bovine adenovirus-3 protein VIII (BAdV-3) to modulate cellular mRNA translation at later stages of BAdV-3 infection (31). Subunit 1 of the eIF3 is one of the proteins interacting with ISG15 to regulate defense responses against infection (32). Moreover, the ubiquitin-like protein ISG15 in cluster D exhibits antibacterial and antiviral activities in marine fish (33, 34).

Skin microbiota (used as environmental biomarker) and the four potential taxonomic microbial biomarkers were identified in the epithelial mucus and feces of *Colossoma macropomum* (35). The host may recruit and regulate the environmental bacteria to shape specific skin microbes, and the interaction between the hosts and their skin microorganisms promotes their coevolution (36). The alpha diversity analysis showed that the skin bacterial structure of the resistant family was different from that of the susceptible family, probably due to the differential expression of the host’s skin proteins. Previous research found four types of pattern recognition receptors, including Toll-like receptors, NOD-like receptors, C-type lectins receptors, and peptidoglycan recognition proteins, associated with disease resistance in fish (37). In the present study, protein-microbial interaction networks identified ten DEPs with the highest number of OTUs, which play important roles in protecting fish from pathogenic bacteria. IGH, BCL10, and PBX were associated with the function of B lymphocytes. The IGH included IGM, IGD and IGT, among which IGM and IGT were involved in the microbiota homeostasis in the mucosal-associated lymphoid tissues of teleost fish (14, 38). IFI35, a member of IFN-induced genes in innate immunity, exhibited antiviral activity and was shown to interact with red-spotted grouper nervous necrosis virus coat protein (39, 40). Micorbiota interaction with BCKDK, HEXO2, RPL3L, CIRBP, ALKBH7, and POLDIP3 had not been reported before.

In summary, the DEPs in the skin of the resistant and susceptible *C. semilaevis* families were identified via iTRAQ analysis and aided the discovery of the molecular mechanisms of fish skin immunity involving the microbial community of marine fish. Therefore, these proteins might be used as molecular markers for disease resistance in fish; however further studies are required to determine their antibacterial roles.

## Data availability statement

The original contributions presented in the study are publicly available. This data can be found here: ProteomeXchange PRJNA1051358 (https://www.ebi.ac.uk/pride/archive/projects/PXD047789) and NCBI BioProject PRJNA1051358 (https://www.ncbi.nlm.nih.gov/bioproject/PRJNA1051358).

## Ethics statement

The animal studies were approved by Yellow Sea Fisheries Research Institute. The studies were conducted in accordance with the local legislation and institutional requirements. Written informed consent was obtained from the owners for the participation of their animals in this study.

## Author contributions

LW: Project administration, Writing – review & editing, Writing – original draft, Software, Methodology, Funding acquisition, Formal Analysis. MT: Writing – original draft, Methodology, Investigation. SC: Writing – review & editing, Funding acquisition.
