# Supplementary material for: Differentially expressed proteins and microbial communities of the skin regulate disease resistance to Chinese tongue sole (Cynoglossus semilaevis)
Source: Front Immunol. 2024 Apr 22;15:1352469. doi: 10.3389/fimmu.2024.1352469 (PMC11071164; doi:10.3389/fimmu.2024.1352469)
Supplement: Supplementary file 1 [file Table_1.docx]

Table S1. The protein ID, the specific primers of these proteins and their related operational taxonomic units (OTUs).

| No. | Protein ID | Protein Name | Abbreviation | Primers in the qRT-PCR | Related OTUs |
| --- | --- | --- | --- | --- | --- |
| 1 | XP_024920185.1 | uncharacterized protein LOC103392960 isoform X47 | IGH | F: CATCTCCAGAGACAACAGCCAA  R: GAGGTAGTCCCCAGTTATTCGAG | 34 OTUs: OTU12, OTU4, OTU93, OTU49, OTU10, OTU24, OTU19, OTU72, OTU61, OTU33, OTU36, OTU130, OTU145, OTU74, OTU131, OTU113, OTU222, OTU325, OTU87, OTU322, OTU123, OTU190, OTU85, OTU196, OTU160, OTU457, OTU170, OTU442, OTU527, OTU315, OTU236, OTU136, OTU168, OTU188 |
| 2 | XP_024921841.1 | B-cell lymphoma/leukemia 10 | BCL10 | F: TAGCATGGCAAGTGACCTCC  R: TACTGAGGGCACATTGAGCG | 23 OTUs: OTU9, OTU12, OTU93, OTU49, OTU25, OTU88, OTU130, OTU254, OTU97, OTU74, OTU131, OTU325, OTU128, OTU69, OTU180, OTU262, OTU626, OTU141, OTU527, OTU77, OTU202, OTU507, OTU374 |
| 3 | XP_024922107.1 | pre-B-cell leukemia transcription factor 1 isoform X2 | PBX2 | F: CCTAACTCGGCAGGTGGCTG  R: TGTGTTGATGCCCTGTGGAC | 24 OTUs: OTU9, OTU12, OTU93, OTU49, OTU10, OTU21, OTU24, OTU19, OTU26, OTU25, OTU36, OTU88, OTU34, OTU130, OTU254, OTU56, OTU131, OTU325, OTU109, OTU69, OTU626, OTU141, OTU527, OTU507 |
| 4 | XP_024920471.1 | interferon-induced 35 kDa protein isoform X3 | IFI35 | F: TAGAGGCCGAGTACCACGAT  R: TCTGTGGTTTCTGAAAGTCGGA | 15 OTUs: OTU49, OTU19, OTU88, OTU130, OTU145, OTU74, OTU131, OTU325, OTU128, OTU180, OTU196, OTU527, OTU202, OTU374, OTU171 |
| 5 | XP_024921174.1 | 3-methyl-2-oxobutanoate dehydrogenase [lipoamide] kinase, mitochondrial | BCKDK | F: GATGTGTCTGCGGAAAAGGC  R: CGATTCGTACAGGCAGCTCT | 19 OTUs: OTU9, OTU25, OTU72, OTU33, OTU425, OTU60, OTU56, OTU97, OTU87, OTU123, OTU367, OTU69, OTU157, OTU160, OTU373, OTU236, OTU609, OTU363, OTU422 |
| 6 | XP_024921204.1 | hexokinase-2-like | HEXO2 | F: GTCAGATCTGTGTGGTCCCC  R: GTCACCGAGACATGAAGCGA | 25 OTUs: OTU9, OTU12, OTU4, OTU93, OTU49, OTU10, OTU21, OTU24, OTU19, OTU26, OTU61, OTU33, OTU36, OTU88, OTU34, OTU130, OTU145, OTU131, OTU325, OTU109, OTU69, OTU196, OTU527, OTU202, OTU171 |
| 7 | XP_024920552.1 | 60S ribosomal protein L3-like isoform X2 | RPL3L | F: CACACCTCTCGCAAGTCCAA  R: AGTGGCCCCATAAACGCTC | 17 OTUs: OTU12, OTU4, OTU93, OTU49, OTU19, OTU61, OTU88, OTU130, OTU145, OTU74, OTU131, OTU325, OTU196, OTU527, OTU202, OTU171, OTU427 |
| 8 | XP_024922086.1 | cold-inducible RNA-binding protein A isoform X3 | CIRBP | F: TGGCAAAGGTGGTGGACAAT  R: CACCACCTCCGAAGTCTCTC | 15 OTUs: OTU49, OTU25, OTU88, OTU425, OTU130, OTU97, OTU180, OTU262, OTU360, OTU77, OTU104, OTU374, OTU530, OTU640, OTU223 |
| 9 | XP_024921175.1 | alpha-ketoglutarate-dependent dioxygenase alkB homolog 4 | ALKBH7 | F: GGTTCACAGACTGCGCTAGA  R: CCGTCATTACTGTCAGGGGG | 18 OTUs: OTU9, OTU25, OTU88, OTU425, OTU56, OTU97, OTU153, OTU69, OTU260, OTU79, OTU262, OTU360, OTU202, OTU104, OTU530, OTU640, OTU384, OTU223 |
| 10 | XP_024920319.1 | polymerase delta-interacting protein 3 isoform X1 | POLDIP3 | F: TCCCAACAGCACAATCAAGC  R: CTTGGTTCCTACACCCCGTC | 18 OTUs: OTU9, OTU25, OTU88, OTU425, OTU56, OTU97, OTU153, OTU69, OTU260, OTU79, OTU262, OTU360, OTU202, OTU104, OTU530, OTU640, OTU384, OTU223 |

Table S2. OTUs list

| #OTU ID | Taxonomy (k, Kingdom; p, Phylum; c, Class; o, Order; f, Family; g, Genus; s, Species ) |
| --- | --- |
| OTU4 | k__Bacteria; p__Proteobacteria; c__Gammaproteobacteria; o__Pseudomonadales; f__Moraxellaceae; g__Enhydrobacter; s__Moraxella_osloensis |
| OTU9 | k__Bacteria; p__Proteobacteria; c__Gammaproteobacteria; o__Pseudomonadales; f__Pseudomonadaceae; g__Pseudomonas; s__Pseudomonas_poae |
| OTU10 | k__Bacteria; p__Proteobacteria; c__Gammaproteobacteria; o__Xanthomonadales; f__Xanthomonadaceae; g__Luteimonas; s__ultramicrobacterium_str._DY01 |
| OTU12 | k__Bacteria; p__Proteobacteria; c__Gammaproteobacteria; o__Enterobacterales; f__Alteromonadaceae; g__Rheinheimera; s__Pararheinheimera_soli |
| OTU19 | k__Bacteria; p__Bacteroidota; c__Bacteroidia; o__Flavobacteriales; f__Flavobacteriaceae; g__Flavobacterium; s__Hydra_vulgaris |
| OTU24 | k__Bacteria; p__Bacteroidota; c__Bacteroidia; o__Flavobacteriales; f__Weeksellaceae; g__Chryseobacterium; s__Kaistella_anthropi |
| OTU25 | k__Bacteria; p__Proteobacteria; c__Alphaproteobacteria; o__Sphingomonadales; f__Sphingomonadaceae; g__Erythrobacter; s__Qipengyuania_flava |
| OTU33 | k__Bacteria; p__Bacteroidota; c__Bacteroidia; o__Flavobacteriales; f__Weeksellaceae; g__Chryseobacterium; s__Epilithonimonas_hominis |
| OTU36 | k__Bacteria; p__Bacteroidota; c__Bacteroidia; o__Flavobacteriales; f__Flavobacteriaceae; g__Flavobacterium; s__Flavobacterium_tegetincola |
| OTU49 | k__Bacteria; p__Proteobacteria; c__Gammaproteobacteria; o__Burkholderiales; f__Oxalobacteraceae; g__Massilia; s__Massilia_eurypsychrophila |
| OTU61 | k__Bacteria; p__Proteobacteria; c__Gammaproteobacteria; o__Pseudomonadales; f__Moraxellaceae; g__Alkanindiges; s__Alkanindiges_sp._JJ005 |
| OTU69 | k__Bacteria; p__Proteobacteria; c__Gammaproteobacteria; o__Burkholderiales; f__Comamonadaceae; g__Limnohabitans; s__beta_proteobacterium_BIWA16 |
| OTU72 | k__Bacteria; p__Proteobacteria; c__Alphaproteobacteria; o__Caulobacterales; f__Caulobacteraceae; g__Brevundimonas; s__Brevundimonas_bullata |
| OTU74 | k__Bacteria; p__Bacteroidota; c__Bacteroidia; o__Flavobacteriales; f__Weeksellaceae; g__Chryseobacterium; s__Chryseobacterium_formosense |
| OTU77 | k__Bacteria; p__Firmicutes; c__Bacilli; o__Lactobacillales; f__Lactobacillaceae; g__Pediococcus; s__Pediococcus_acidilactici |
| OTU85 | k__Bacteria; p__Proteobacteria; c__Alphaproteobacteria; o__Rhodobacterales; f__Rhodobacteraceae; g__Paracoccus; s__Paracoccus_zhejiangensis |
| OTU87 | k__Bacteria; p__Actinobacteriota; c__Actinobacteria; o__Micrococcales; f__Intrasporangiaceae; g__Knoellia; s__Knoellia_sp. |
| OTU88 | k__Bacteria; p__Proteobacteria; c__Gammaproteobacteria; o__Enterobacterales; f__Aeromonadaceae; g__Aeromonas; s__Aeromonas_caviae |
| OTU93 | k__Bacteria; p__Proteobacteria; c__Gammaproteobacteria; o__Burkholderiales; f__Oxalobacteraceae; g__Massilia; s__Massilia_aurea |
| OTU97 | k__Bacteria; p__Deinococcota; c__Deinococci; o__Deinococcales; f__Deinococcaceae; g__Deinococcus; s__Deinococcus_aquaticus |
| OTU113 | k__Bacteria; p__Proteobacteria; c__Alphaproteobacteria; o__Rhizobiales; f__Rhizobiaceae; g__Allorhizobium_Neorhizobium_Pararhizobium_Rhizobium; s__Rhizobium_leguminosarum |
| OTU123 | k__Bacteria; p__Proteobacteria; c__Alphaproteobacteria; o__Rhizobiales; f__Beijerinckiaceae; g__Bosea; s__Bosea_sp._B0.09_49 |
| OTU128 | k__Bacteria; p__Firmicutes; c__Bacilli; o__Exiguobacterales; f__Exiguobacteraceae; g__Exiguobacterium; s__Exiguobacterium_sibiricum |
| OTU130 | k__Bacteria; p__Proteobacteria; c__Gammaproteobacteria; o__Xanthomonadales; f__Xanthomonadaceae; g__Stenotrophomonas; s__Stenotrophomonas_rhizophila |
| OTU131 | k__Bacteria; p__Proteobacteria; c__Gammaproteobacteria; o__Burkholderiales; f__Comamonadaceae; g__Acidovorax; s__Acidovorax_defluvii |
| OTU136 | k__Bacteria; p__Proteobacteria; c__Alphaproteobacteria; o__Sphingomonadales; f__Sphingomonadaceae; g__Sphingobium; s__Sphingobium_algorifonticola |
| OTU141 | k__Bacteria; p__Proteobacteria; c__Gammaproteobacteria; o__Xanthomonadales; f__Xanthomonadaceae; g__Lysobacter; s__Lysobacter_sp. |
| OTU145 | k__Bacteria; p__Proteobacteria; c__Gammaproteobacteria; o__Burkholderiales; f__Comamonadaceae; g__Delftia; s__Delftia_tsuruhatensis |
| OTU160 | k__Bacteria; p__Bacteroidota; c__Bacteroidia; o__Sphingobacteriales; f__Sphingobacteriaceae; g__Pedobacter; s__Pedobacter_boryungensis |
| OTU168 | k__Bacteria; p__Firmicutes; c__Bacilli; o__Bacillales; f__Marinococcaceae; g__Salsuginibacillus; s__Salsuginibacillus_kocurii |
| OTU170 | k__Bacteria; p__Proteobacteria; c__Gammaproteobacteria; o__Burkholderiales; f__Rhodocyclaceae; g__Dechloromonas; s__Dechloromonas_sp._JM |
| OTU180 | k__Bacteria; p__Proteobacteria; c__Gammaproteobacteria; o__Burkholderiales; f__Burkholderiaceae; g__Limnobacter; s__Limnobacter_thiooxidans |
| OTU188 | k__Bacteria; p__Bacteroidota; c__Bacteroidia; o__Flavobacteriales; f__Flavobacteriaceae; g__Flavobacterium; s__Flavobacterium_sp._R_21935 |
| OTU190 | k__Bacteria; p__Proteobacteria; c__Alphaproteobacteria; o__Sphingomonadales; f__Sphingomonadaceae; g__Sphingopyxis; s__Sphingopyxis_nepalensis |
| OTU196 | k__Bacteria; p__Actinobacteriota; c__Actinobacteria; o__Propionibacteriales; f__Nocardioidaceae; g__Nocardioides; s__Nocardioides_currus |
| OTU202 | k__Bacteria; p__Firmicutes; c__Bacilli; o__Lactobacillales; f__Enterococcaceae; g__Enterococcus; s__Enterococcus_faecium |
| OTU222 | k__Bacteria; p__Firmicutes; c__Negativicutes; o__Veillonellales_Selenomonadales; f__Selenomonadaceae; g__Megamonas; s__Megamonas_funiformis |
| OTU236 | k__Bacteria; p__Bacteroidota; c__Bacteroidia; o__Cytophagales; f__Spirosomaceae; g__Arcicella; s__Arcicella_rosea |
| OTU254 | k__Bacteria; p__Proteobacteria; c__Gammaproteobacteria; o__Pseudomonadales; f__Halomonadaceae; g__Halomonas; s__Halomonas_muralis |
| OTU262 | k__Bacteria; p__Verrucomicrobiota; c__Verrucomicrobiae; o__Chthoniobacterales; f__Chthoniobacteraceae; g__Candidatus_Udaeobacter; s__Candidatus_Udaeobacter_copiosus |
| OTU315 | k__Bacteria; p__Firmicutes; c__Clostridia; o__Lachnospirales; f__Lachnospiraceae; g__[Eubacterium]_hallii_group; s__Lachnospiraceae_bacterium_MC_36 |
| OTU322 | k__Bacteria; p__Proteobacteria; c__Gammaproteobacteria; o__Burkholderiales; f__Comamonadaceae; g__Comamonas; s__Comamonas_terrigena |
| OTU325 | k__Bacteria; p__Proteobacteria; c__Gammaproteobacteria; o__Burkholderiales; f__Oxalobacteraceae; g__Massilia; s__Massilia_dura |
| OTU374 | k__Bacteria; p__Bacteroidota; c__Bacteroidia; o__Bacteroidales; f__Tannerellaceae; g__Parabacteroides; s__Parabacteroides_distasonis |
| OTU442 | k__Bacteria; p__Bacteroidota; c__Bacteroidia; o__Flavobacteriales; f__Flavobacteriaceae; g__Flavobacterium; s__Flavobacterium_cutihirudinis |
| OTU457 | k__Bacteria; p__Proteobacteria; c__Alphaproteobacteria; o__Rhodobacterales; f__Rhodobacteraceae; g__Defluviimonas; s__Defluviimonas_aquaemixtae |
| OTU507 | k__Bacteria; p__Proteobacteria; c__Gammaproteobacteria; o__Enterobacterales; f__Yersiniaceae; g__Serratia; s__Serratia_liquefaciens |
| OTU527 | k__Bacteria; p__Proteobacteria; c__Gammaproteobacteria; o__Enterobacterales; f__Shewanellaceae; g__Shewanella; s__Shewanella_baltica |
| OTU626 | k__Bacteria; p__Proteobacteria; c__Gammaproteobacteria; o__Pseudomonadales; f__Moraxellaceae; g__Acinetobacter; s__Acinetobacter_bereziniae |
